# Effects of Heating Rates on Microstructural Evolution of Hot Extruded 7075 Aluminum Alloy in the Semi-Solid State and Thixotropic Deformation Behavior

**DOI:** 10.3390/ma16186145

**Published:** 2023-09-10

**Authors:** Guochao Gu, Ruifen Li, Lixin Xiang, Guiyong Xiao, Yupeng Lu

**Affiliations:** 1 Key Laboratory for Liquid-Solid Structural Evolution and Processing of Materials, Ministry of Education, Shandong University, Jinan 250061, China; 202034221@mail.sdu.edu.cn (L.X.); xiaoguiyong@sdu.edu.cn (G.X.); 2 School of Materials Science and Engineering, Shandong University, Jinan 250061, China; 3 Suzhou Institute, Shandong University, Suzhou 215123, China; 4 Shandong Institute for Product Quality Inspection, Jinan 250102, China; liruifen2016@163.com

**Keywords:** 7075 aluminum alloy, grain coarsening, thixo-compression, heating rate

## Abstract

The non-dendritic microstructure plays a crucial role in determining the rheological properties of semi-solid alloys, which are of the utmost importance for the successful industrial application of the thixoforging process. To further understand the impact of the reheating process on the evolution of microstructure and thixotropic deformation behavior in the semi-solid state, a hot extruded and T6 treated 7075 aluminum alloy was reheated to the selected temperature ranges using varying heating rates. Subsequently, thixo-compression tests were performed. The study found that during reheating and isothermal holding, the elongated microstructure of the as-supplied alloy can transform into equiaxed or spherical grains. The presence of recrystallized grains was found to be closely linked to the penetration of the liquid phase into the recrystallized grain boundaries. Furthermore, it was observed that higher heating rates resulted in smaller grain sizes. The thixotropic flow behavior of the alloy with various microstructures was analyzed using the true stress–strain curves obtained by thixo-compression experiments, which exhibited three stages: a rapid increase in true stress to a peak value, followed by a decrease in true stress and a steady stress until the end of compression. The stress fluctuated with strain during the formation of the slurry at a strain rate of 10 s^−1^, indicating the significant role of strain rate in material flow during semisolid formation.

## 1. Introduction

The 7XXX series age-hardening alloys are known for their high specific strengths in the aluminum alloy category, making them suitable for various applications that demand lightweight materials [1]. The high strength of Al-Zn-Mg-Cu alloys can be achieved through the formation of reinforcing precipitates during a controlled heat treatment [2]. The 7075 aluminum alloy, which primarily contains zinc as the main alloying element, is recognized as the strongest grade of aluminum alloy, making it highly suitable for aerospace and structural applications. The main strengthening phase for 7075 and other alloys with Zn:Mg ratios between 1:2 and 1:3 is the fine MgZn_2_ precipitates formed at aging temperatures below 200 °C. In addition, chromium-containing dispersed particles, specifically Al_12_Mg_2_Cr and Al_18_Mg_3_Cr_2_ (E-phase), precipitate from the solid solution during homogenization and solution heat treatment. These particles significantly influence the recrystallization process and hinder grain growth. Impurities such as iron and silicon can result in the formation of inclusions in 7075 aluminum alloy, which can have a significant negative impact on various mechanical properties, including fracture toughness and fatigue strength [3]. Whilst conventional forging is a viable method for shaping 7075 aluminum alloy and enhancing its mechanical properties through structural refinement, it often requires expensive machining and results in substantial material wastage. Consequently, there is a considerable practical interest in achieving a near-net shape for different alloys.

The semi-solid forming process of aluminum alloys, which offers advantages over both conventional casting and solid forming processes, is widely recognized as a near-net shape process [4]. By taking advantage of the rheological behavior of the non-dendritic microstructure in the semi-solid state, the potential applications of this technique for producing complex aluminum parts with thinner sidewalls and narrower dimensional tolerances have been investigated by many researchers [5]. Generally, this process can be divided into two main routes: thixo- and rheo-routes. The rheoforming processes with low solid fraction have already been commercialized for Al-Si based aluminum alloys, because of their good fluidity and formability [4,5]. However, the high-strength wrought aluminum alloys for thixoforming at high solid fraction need to be further investigated for further commercialization [6]. Thixoforging is a branch of semi-solid metal forming. It involves reheating the billet to achieve a semi-solid state at a high solid fraction with a desired microstructure that exhibits thixotropic behavior. Subsequently, the billet is compelled into the die. In order to obtain thixotropic behavior, it is necessary that the starting material has an equiaxed microstructure [7]. Generally, the thixotropic behavior of the semi-solid billets improves as the grains become smaller and globular. Therefore, further investigation should be conducted to explore the relationship between thixotropic behavior and microstructural characteristics in order to achieve a successful thixoforging process.

The required near-spheroidal microstructure can be obtained by various semi-solid billet preparation routes, which can be generally divided into the liquid metal route and the solid-state route. They were summarized and introduced in detail by Nafisi and Ghomashchi [5]. Czerwinski [8] also detailed various methods and their differences for semi-solid billet preparation. Solid-routes such as recrystallization, the partial melting (RAP) process [9], and the strain induced melt activation (SIMA) process [10] have been widely investigated and applied in the preparation of many semi-solid metal alloys, such as 319 [11], 7075 [12], Mg-10Gd-3Y-1Zn-0.4Zr [13], etc. The 7075 aluminum alloys prepared by the SIMA [14] and RAP [15] routes have been investigated, and the results demonstrated that a significantly more uniform and finer equiaxed microstructure is feasible compared to those prepared through liquid routes. The two routes are similar but distinct. The SIMA method employs a high-temperature working process above the recrystallization temperature. Conversely, the RAP method involves working at temperatures lower than the recrystallization temperature. Jiang et al. [14] compared the grain coarsening rate of 7075 prepared by the RAP and SIMA routes and concluded that there was no significant difference in the coarsening rate of the alloy prepared by these two routes.

Both of these methods induce severe plastic deformation (SPD) in the material for the purpose of attaining a substantial dislocation density, which serves as the driving force for the subsequent recrystallization during the reheating process [16]. During the reheating process, recrystallization occurs, and the liquid penetrates the recrystallized boundaries, resulting in near-spheroidal grains [3]. Therefore, some pre-deformation methods such as equal channel angular pressing (ECAP) [17] and repetitive upsetting extrusion (RUE) [18] have also been used for the preparation of semi-solid slurries. Since some alloys are always supplied in the deformed state (rolled or extruded) with equiaxed grains, the required near-spheroidal microstructure could be obtained by recrystallization and coarsening during the direct reheating process and isothermal treatment, leading to a great reduction in the cost. A short process involving heating the mass-supplied hot-deformed wrought alloys directly to fabricate semi-solid billets was proposed, namely wrought aluminum directly semi-solid isothermal treatment (WADSSIT) [6].

The effectiveness of these solid-routes in achieving a spherical structure depends on the rate of pre-deformation and recrystallization behavior. Bolouri et al. [19] stated that the average grain size of 7075 prepared by the SIMA method decreases gradually with an increase in the compression rate. Meanwhile, Sang et al. [20] who investigated the impact of cold working and heating conditions on the microstructure of this alloy in the SIMA process, indicated that a minimum of 50% cold working was required to achieve a uniform microstructure during semi-solid heating. Recently, Shabestari et al. [21] investigated the effect of pre-deformation rate on the SIMA process of Al-10.5%Si-3Cu-0.2Mg alloy. The results showed that as the pre-deformation increased from 10% to 20%, the average grain size exhibited a significant decrease of 45.8%, while the average shape factor showed a notable increase of 55.8%. Undoubtedly, microstructure refinement could also be realized by recrystallization. The recrystallization behavior of 2A14 [22], 6A02 [23], and 7075 aluminum [3,24] alloys in the semi-solid state was investigated, with a focus on the effects of the isothermal treatment stage. However, the temperature rising process could provide valuable parameters for the subsequent isothermal stage. While the effect of heating rates on recrystallization in the solid ranges has been studied [25], its impact at the semi-solid temperature has seldom been investigated.

Furthermore, it has been observed that extensive coarsening can negatively impact the material flows and mechanical properties of thixoforged parts. Previous investigations focused on the recrystallization and coarsening of the as-extruded 7075 alloy. However, there is a lack of studies in the literature that consider the combined effects of heating rate on the microstructural evolution and thixoformability. In addition, it is of interest to prepare the semi-solid billets by directly reheating the hot deformed 7075 alloys in order to reduce the manufacturing cost. The present study aimed to investigate the impact of the reheating rate on recrystallization and grain size in the semi-solid temperature range of 7075 aluminum alloy. The study evaluated the effects of reheating parameters (heating rate, holding time, temperature, etc.) on the microstructure evolution during partial remelting of as-extruded 7075. In addition, isothermal thixo-compression tests were conducted to discuss the effects of process parameters and microstructures on the thixotropic deformation behavior throughout the thixo-compression process.

## 2. Experimental Procedures

### 2.1. Material

The 7075 aluminum alloy utilized in this study was supplied by Chinalco Southwest Aluminum (Group) Co., Ltd., Chongqing, China. The material was hot extruded (with an extrusion ratio of 16) and T6 treated. The chemical composition of the alloy was determined using an X-ray fluorescence spectrometer, as shown in Table 1. 

**Figure 1:**
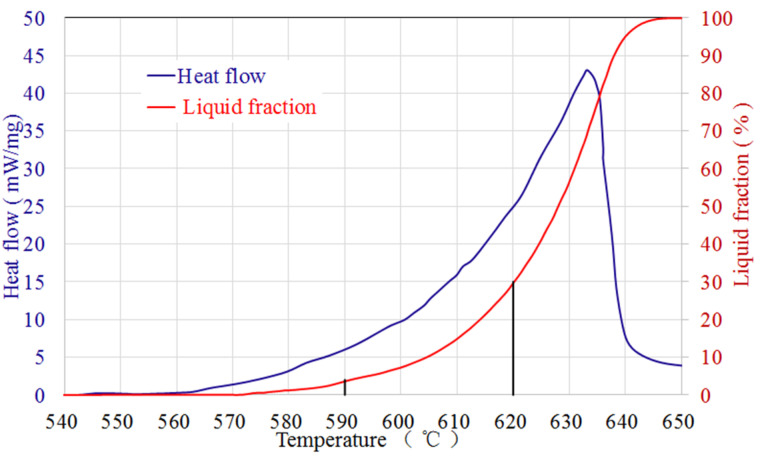
Results of DSC tests. Heat flow versus temperature (in blue) and liquid fraction as a function of temperature (in red) of 7075 aluminum alloy.

A differential scanning calorimeter (DSC, SDT-Q600, Thermo Electron Corporation, Franklin, MI, USA) was used to obtain the heat flow-temperature curve. Samples (~5 mg) of 0.5 mm in thickness were subjected to heating from ambient temperature to 700 °C. The heating rate was set at 10 °C/min within an alumina sample holder under a nitrogen atmosphere. Subsequently, the samples were cooled down to room temperature at the same rate. Throughout the experiments, thermocouples were utilized to meticulously monitor both the heat flow and temperature, thus enabling heating and cooling curves to be acquired. By integrating the data obtained from the DSC tests, we successfully calculated the correlation between the temperature and the liquid volume fraction, as illustrated in Figure 1 [6]. Based on the DSC curve, the solidus and liquidus temperatures were ~545 °C and ~652 °C, respectively. Additionally, it was found that the material exhibits a relatively low temperature sensitivity below 620 °C. As a result, further investigations were carried out to explore the microstructure evolution and thixotropic deformation characteristics of the material at a temperature range from 590 to 620 °C, in order to achieve the required thixotropic behavior and avoid the strong ‘elephant foot’ phenomenon caused by a high liquid fraction.

### 2.2. Reheating Process

Semi-solid billets of the 7075 aluminum alloy were obtained by the direct partial re-melting process. In order to have a homogeneous temperature distribution throughout the sample, samples with dimension of Ф 8 mm × 12 mm were machined from as-extruded rods. In the low heating rate experiments, the samples underwent a gradual heating process, starting from ambient temperature and reaching the intended temperatures using a resistance furnace. The heating rate averaged around 15 °C/min. After being held for different durations (0 min, 5 min, 15 min, 30 min, and 60 min), the samples were immediately quenched in water. On the other hand, a Gleeble 1500 thermomechanical simulator (Dynamic System, Inc. Washington, America) was used to heat the samples to the desired temperatures at a heating rate of 600 °C/min, and then quenched after holding for 1 min at the room temperature. For temperature measurement, a hole of 2 mm in diameter and 2 mm in depth was drilled in the center of each sample to accommodate a K-type thermocouple (with an error of ±2 °C). 

### 2.3. Isothermal Compression Test

**Figure 2:**
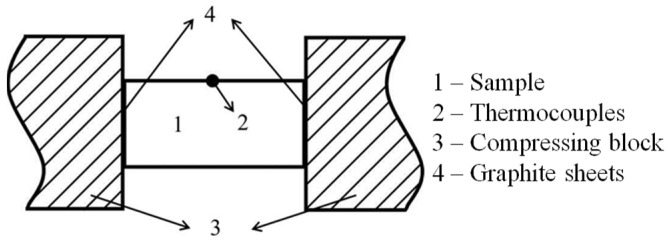
Schematic diagram of isothermal compression tests for rapid reheating samples.

To investigate the impacts of microstructure on thixotropic deformation behavior, isothermal compression tests were conducted. The rapid reheated samples were compressed horizontally using the Gleeble 1500 thermomechanical simulator, while the samples slowly reheated by the resistance furnace were transferred to a high-temperature compression test machine equipped with zero-force clamping and compressed. Figure 2 shows the schematic diagram of isothermal compression. The samples were subjected to thixo-compression until a total strain of 0.6 was achieved, with varying strain rates (10^−3^ s^−1^, 10^−2^ s^−1^, 10^−1^ s^−1^, 1 s^−1^, and 10 s^−1^). Graphite lubrication was applied to minimize friction between the sample and clamps. Furthermore, yttrium oxide was coated on both ends of the samples to prevent the sample from sticking during compression. After thixo-compression, the samples were immediately quenched in water to preserve the microstructure. Compression tests were conducted under the same conditions to ensure experimental accuracy and repeatability. Subsequently, the samples were sectioned along the compression direction for metallographic observations.

### 2.4. Metallographic Characterization

The samples at various states were sectioned along the longitudinal direction and then mounted in Bakelite for microstructure observation preparations. They were ground with SiC abrasive paper (P800–P1200 grit), polished with diamond spry (2.5 μm), and finally etched with Keller’s agent (1 mL HF, 1.5 mL HCl, 2.5 mL HNO_3_, and 95 mL H_2_O) for about 15 s. The microstructures were observed by an optical micrograph (Zeiss Axio Lab. A1, Carl Zeiss AG, Oberkochen, Germany) and a scanning electronic microscope (JSM-7610F, JEOL Ltd., Tokyo, Japan). The element distribution of reheated samples was determined by EDS analysis equipped in the SEM (JSM-7610F). The average grain size was quantitatively calculated by the Image Pro Plus software. More than 500 grains for each sample were measured for the calculation. Equation (1), used for the average diameter *d* calculation, is as follows:

(1) $$d=\frac{\sum_{i=1}^{N}\sqrt{4A_{i}/\pi }}{N}$$

where $A_{i}$ is the area of the grain i, *N* is the number of grains.

## 3. Results

### 3.1. Microstructure Evolution and Element Distribution during Reheating

**Figure 3:**
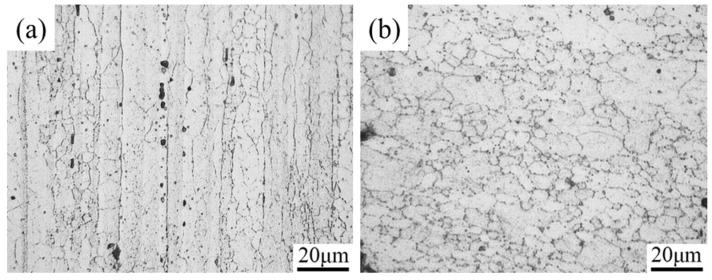
Microstructure of as-received 7075 aluminum alloy on: (**a**) longitudinal section, (**b**) transversal section.

Figure 3 presents the optical micrographs of the hot-extruded 7075 aluminum alloy observed along longitudinal (Figure 3a) and transversal (Figure 3b) sections. The microstructure consists of α-Al phase (white grey) with intermetallic particles (dark grey). The eutectic particles are mainly found along the grain boundaries and also partially exist in the α-Al phase. The elongated grains (Figure 3a) running parallel to the manufacturing working direction are typical for hot working materials. In the transversal section, the microstructure exhibits a morphology of like equiaxed grains (Figure 3b). The microstructure of the raw material is heterogeneous, exhibiting large variations in grain size on both longitudinal and cross sections, indicating partial recrystallization during T6 treatment.

Based on the results of EDS analysis (Table 2), it is evident that the matrix primarily consists of the α-Al phase (Position A). Additionally, a small amount of Zn, Mg, and Cu were soluted into the matrix. The presence of small spherical particles (Position B) suggests the existence of the Al_2_CuMg phase, which is generally formed in aluminum alloys when Cu content exceeds 1 wt%. The black irregular-shaped particles (Position C) are likely the Mg_2_Si phase. In the 7075 aluminum alloy, Si and Fe elements are present as impurities. The white irregular particles (Positions D, E, and F) represent the second phase, which contains a higher concentration of Cu and Fe elements. Since the atomic radius of Mn and Cr is relatively close to that of Fe, some Fe atoms are replaced by Mn and Cr in solid solution, so the second phase also contains a small amount of Mn and Cr elements.

**Figure 4:**
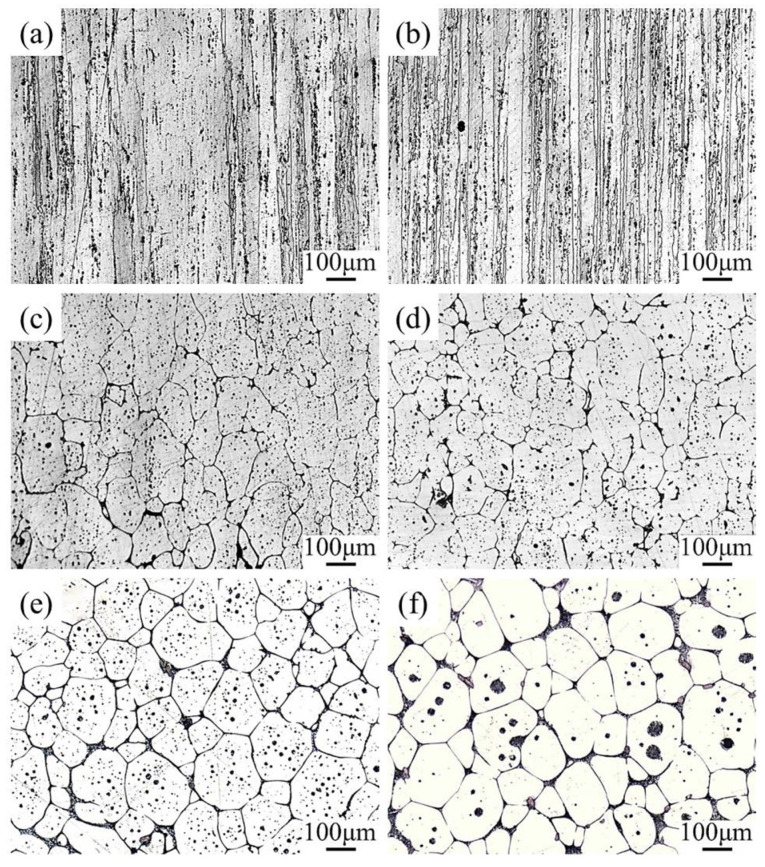
Microstructure of as-received 7075 Al alloy after reheating from room temperature to various predetermined temperatures. (**a**) 450 °C, (**b**) 500 °C, (**c**) 550 °C, (**d**) 570 °C, (**e**) 600 °C, and (**f**) 620 °C.

Figure 4 illustrates the microstructure evolution of the alloy when subjected to reheating at different temperatures (450 °C, 500 °C, 550 °C, 570 °C, 600 °C, and 620 °C) using an electric resistance furnace. The reheating duration was kept constant at 30 min. Similar to the as-received state (Figure 3), the elongated grains were observed along the extrusion direction below 500 °C. However, at 500 °C some recrystallized grains were formed (Figure 4b). When the material was reheated into the semi-solid state, an abrupt emergence of significantly enlarged recrystallized grains was observed (Figure 4c). Additionally, due to the infiltration of low-melting phases, the degree of recrystallization exhibited enhancement, resulting in a more pronounced visibility of the grain boundaries. Furthermore, with increasing reheating temperature, the liquid fraction also increased, and the grains became more spherical, as observed in Figure 4d–f. Moreover, the size of the liquid droplets was larger at higher temperatures (Figure 4f) compared to lower temperatures (Figure 4e), indicating the dissolution of low melting point precipitates. On comparing the micrographs in Figure 4, it can be concluded that the recrystallization degree of the alloy increased with the heating temperature. 

**Figure 5:**
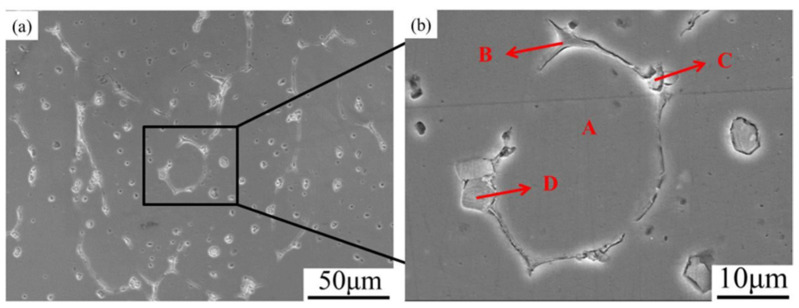
(**a**) SEM micrograph of 7075 Al alloy held at 550 °C for 15 min; (**b**) micrograph of the black square shown in (**a**).

**Figure 6:**
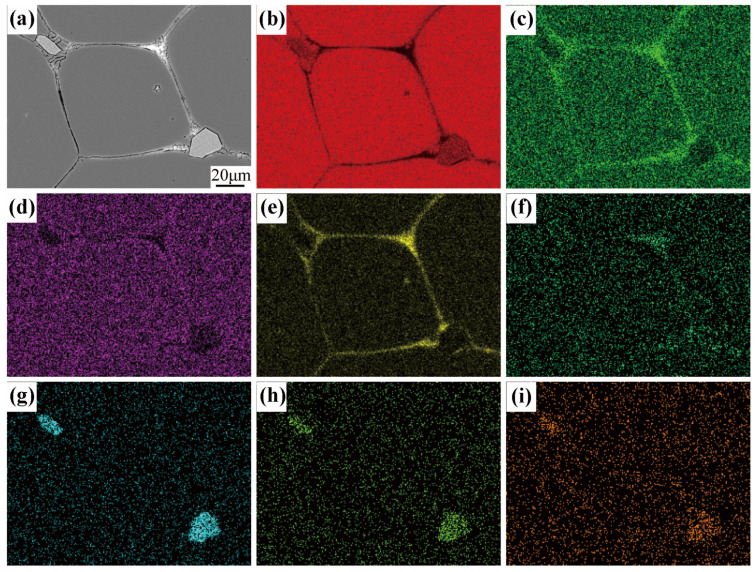
The EDS mapping of semi-solid 7075 Al alloy after holding at 610 °C for 15 min: (**a**) SEM micrograph, (**b**) Al, (**c**) Zn, (**d**) Mg, (**e**) Cu, (**f**) Si, (**g**) Cr, (**h**) Mn, (**i**) Fe.

The SEM micrographs presented in Figure 5 provide confirmation that the formation of liquid stimulates recrystallization in the 7075 aluminum alloy when quenched at 550 °C and soaked for 5 min. At 550 °C, there is a significant change in morphology compared to the initial hot-extruded state. The solid grains are partially surrounded by newly formed precipitates (Point B and C), corresponding to the former liquid phase in the semi-solid state, as well as undissolved precipitates with a square shape (Point D). Table 3 contains a summary of the EDS results for the designated areas indicated in the SEM image. The EDS results of Point B and C reveal a high concentration of Cu and Zn elements, which are known to form low melting point eutectic phases and disperse along the grain boundaries during heating [26]. Moreover, the high concentration of Cu at the grain boundaries leads to a depletion of Cu in the solid grains. Consequently, this results in an elevation in the solid grains’ solidus temperature and a decline in temperature at the grain boundaries. As the heating or soaking time increases, these lower melting point precipitates undergo melting and dispersion along the grain boundaries, penetrating the recrystallized grain boundaries, and ultimately leading to the formation of spheroids. On the other hand, Fe-rich precipitate phases (Point D in Figure 5) as indicated by the EDS analysis remain undissolved and appear predominantly as square particles. According to the findings of Boettinger et al. [27], it was observed that Fe-rich precipitates exhibit elevated solution temperatures and tend to remain stable when kept at temperatures surpassing 600 °C. The undissolved Fe-rich precipitates were still observed even after being reheated to 610 °C and soaked for 15 min, as depicted in Figure 6. The confirmation of the undissolved state of the Fe-rich precipitates is further supported by the EDS mapping. Additionally, noticeable segregation of Cu and Zn at the grain boundaries and the presence of small droplets within the solid grains indicate the presence of a surrounding liquid phase. The element distribution and the undissolved precipitates may result in a low coarsening rate during the isothermal reheating process. During the isothermal treatment in a semi-solid state, the precipitates with a lower melting point will first undergo melting and then disperse along the grain boundaries. The liquid phases present can act as pathways for the diffusion of alloying elements, whereas the undissolved phases containing a high concentration of Fe can limit the extent of grain boundary growth. As a result, the diffusion rate of alloying elements is reduced, and the movement of solute is restricted, ultimately leading to a decrease in the rate at which the grains coarsen. The inhibitory effect of intermetallic particles on the coarsening rate was also demonstrated in the studies conducted by Xiao et al. [24] and Fu et al. [17], providing valuable insights for controlling grain coarsening during the preparation of semi-solid billets.

**Figure 7:**
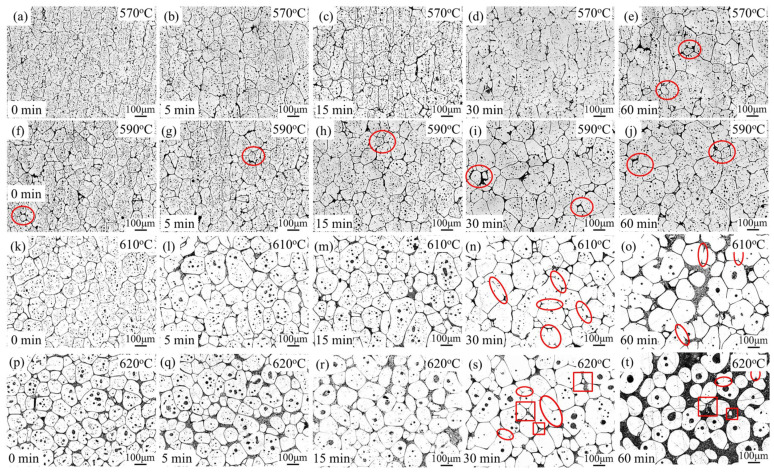
Microstructural evolution of 7075 reheated and then held in the semi-solid state in an electrical furnace: (**a**–**e**) 0, 15, 30, 60 min at 570 °C; (**f**–**j**) 0, 15, 30, 60 min at 590 °C; (**k**–**o**) 0, 15, 30, 60 min at 610 °C; (**p**–**t**) 0, 15, 30, 60 min at 620 °C (longitudinal sections).

The effects of isothermal treatment on the microstructure in the semi-solid state at temperatures ranging from 570–620 °C were investigated, as compared in Figure 7. Different from the as-received state, the microstructure in the semi-solid state consists of solid α-Al grains (white grey), liquid phases (dark grey), and some tiny undissolved precipitate particles (depicted in Figure 6). The liquid phase is distributed at the grain boundaries and in the solid grains as well. Figure 7a–e illustrates the microstructures of the alloy isothermally reheated at 570 °C for various holding times. Initially, the spheroidization phenomenon exhibited unsatisfactory results, as noticeably elongated grains still exist after a 5 min hold (Figure 7a). However, with increasing holding time, the grain shape evolved into an equiaxed shape (Figure 7b,c). After 60 min (Figure 7e), the grain boundaries became smoother and faceted, although the grains still exhibited an irregular shape. This is attributed to the low presence of liquid phase at the grain boundaries. The size of the recrystallized grains exhibits high dispersion, ranging from a few micrometers (as indicated by the red circles) to a hundred micrometers. Additionally, small intragranular islands, believed to be liquid pools, were observed within the solid grains just prior to quenching. The presence of fine liquid droplets is a typical characteristic of semi-solid microstructures and has been reported in various alloys [10,28]. 

Regarding the micrographs shown in Figure 7f–j, heating the deformed sample at 590 °C for different durations results in similar microstructure evolution to those heated at 570 °C. However, there was an improvement in the roundness of the solid grains. When the samples were held at 610 °C and 620 °C for varying durations as shown in Figure 7h–t, it can be observed that the spherical grains were obtained after holding for 5 min, indicating successful spheroidization at higher temperatures. As the holding time increased, the grain boundaries became thicker. Additionally, after undergoing an extended heating procedure, certain small liquid droplets then amalgamate with the larger ones. Consequently, the recently created liquid droplets adopt a more globe-like structure with the aim of diminishing the interfacial energy between the solid and the liquid. 

**Figure 8:**
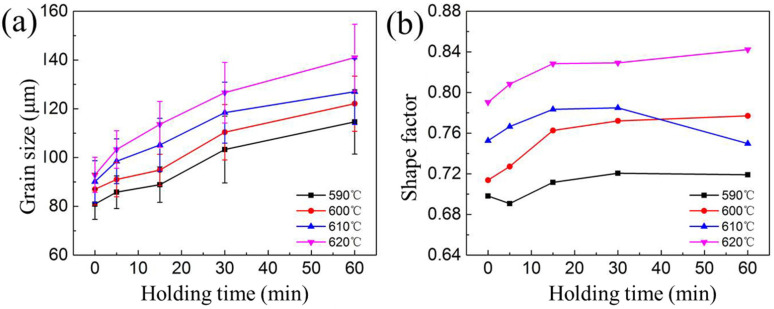
(**a**) Variations of grain size and (**b**) shape factor as a function of different isothermal holding times at 590–620 °C.

It is clear that spheroidization and growth of grains occurred during the isothermal reheating process. Figure 8 illustrates the variations in shape factor and average grain size of the alloy as a function of isothermal temperatures and holding times. The average grain size increases as the temperature rises. During isothermal heating, the microstructure undergoes two main stages. In the first stage, recrystallization and partial remelting occur at low temperatures and for short holding durations, as it was shown in Figure 4 that recrystallization below the solidus temperature is difficult due to the presence of dispersoids. However, when the alloy was heated up into the semi-solid state, the dispersoids, which act as pinning boundaries, start to dissolve into the liquid phase, thereby initiating recrystallization as the boundaries become unpinned [3]. In this stage, grains with high dislocation density are replaced by new grains with lower dislocation density. Subsequently, in the second stage, spheroidization and coarsening of grains occur after the generation of the liquid phase. Other researchers, who analyzed the coarsening behavior of A356 [29] and ZK 60 magnesium alloy [30] during the semi-solid state reported that grain coarsening mainly involves coalescence and Ostwald ripening mechanisms. The presence of liquid at the grain boundary reduces the grain boundary energy. Coalescence occurs between adjacent solid grains, as shown by the red ellipses in Figure 7. Low liquid fraction results in thin liquid films along the grain boundaries. Consequently, a large number of solid grains come into contact with each other. The dominant mechanism for grain growth is coalescence. As the isothermal temperature and holding time increase, a significant number of liquid phases distribute along the grain boundaries, resulting in the formation of discrete solid grains. During this process, small solid grains dissolve (see the red rectangles) while large solid grains continue to grow, resulting in an increase in the average solid grain size. Consequently, the effect of coalescence decreases, and Ostwald ripening becomes dominant.

The coarsening kinetic is expressed by the Lifshitz–Slyozov–Wagner (LSW) relationship (Equation (2)):

(2) $$D^{n}-D_{0}^{n}=kt$$

where in the semi-solid state, volume diffusion-controlled systems exhibit a power exponent of 3, denoted as *n*; *t* represents the isothermal holding time while D and $D_{0}$ signify the final and initial grain sizes, respectively. The coarsening rate constant is denoted as k in this context [29].

**Figure 9:**
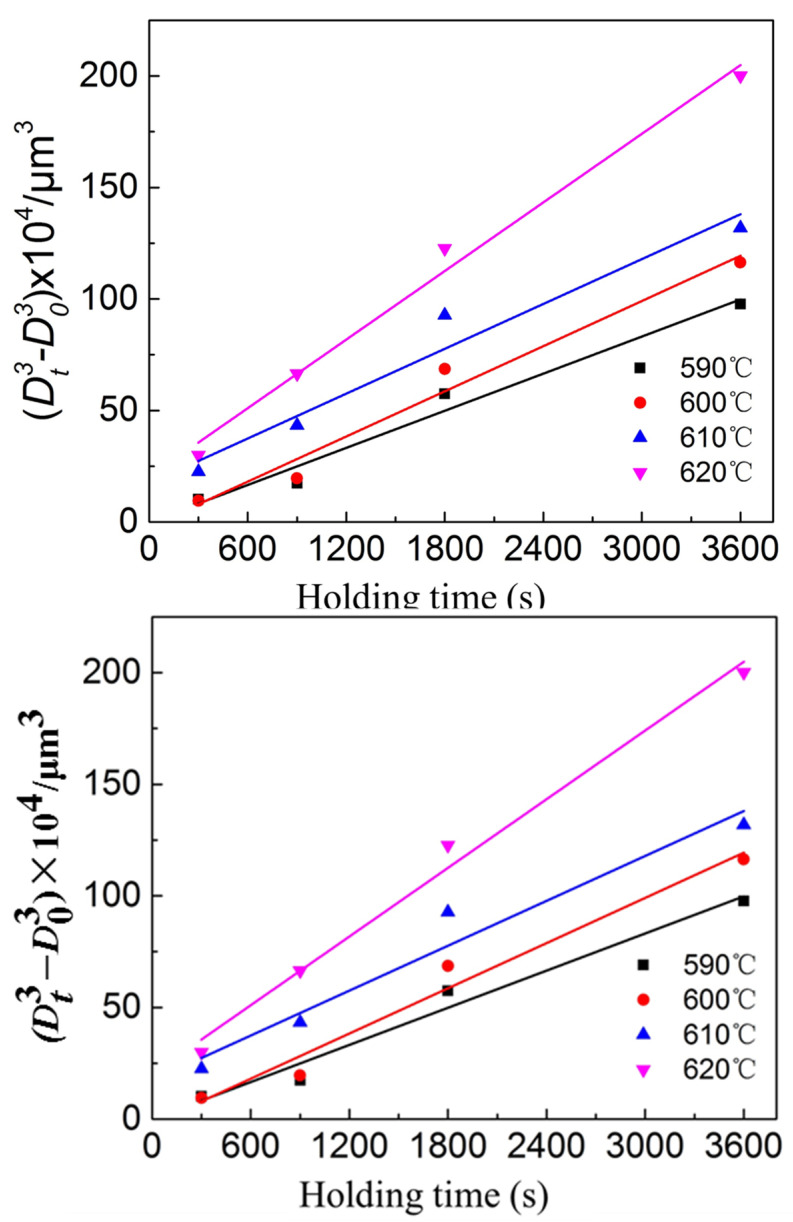
Variations of average grain size as a function of isothermal holding time at heating temperatures of 590–620 °C.

In the current study, we determined the coarsening rate constant (*k*) by fitting an exponential function to the empirical data. In Figure 9, we observe the changes in average grain size over different isothermal holding times for samples subjected to heating temperatures of 590 °C, 600 °C, 610 °C, and 620 °C. Here $D_{0}$ represents the average grain size at a holding time of 5 min. Our findings indicate a strong association between the coarsening kinetics of solid particles during isothermal heating of deformed 7075 samples within the semi-solid temperature range and the LSW equation. The coarsening rate constants at 590 °C, 600 °C, 610 °C, and 620 °C are 277 μm^3^/s, 337 μm^3^/s, 335 μm^3^/s, and 513 μm^3^/s, respectively. In addition, an increase of the holding temperature results in the increase of the coarsening rate constant. Fu et al. [15] used the RAP method to study the coarsening rate constant of 7075 aluminum alloy at 227–327 μm^3^/s (570–605 °C). Binesh et al. [31] used the SIMA method to study the grain growth rate of 7075 aluminum alloy. The growth rate is 345–515 μm^3^/s (600–620 °C), which is close to 277–513 μm^3^/s of 7075 aluminum alloy in this study. In general, increasing the heating temperature leads to an acceleration in the coarsening rate of solid grains. This is because it promotes the development of a fast diffusion path due to an increase in the liquid fraction. Furthermore, Manson-Whitton et al. [32] found that the connection between solid particles results in a decrease of the coarsening rate constant for solid fractions greater than 0.7, which is attributed to a decrease in the solid–liquid interfacial area.

**Figure 10:**
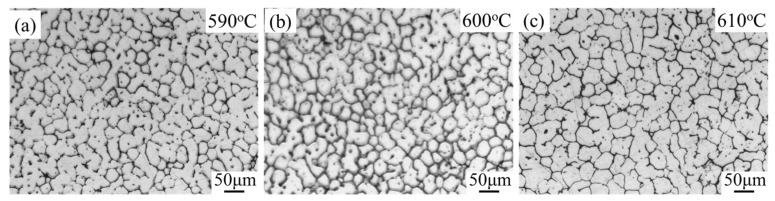
Microstructure evolution of 7075 aluminum alloy after reheating at different temperatures with a heating rate of 600 °C/min. (**a**) 590 °C; (**b**) 600 °C; (**c**) 610 °C.

Figure 10 displays the microstructure of 7075 aluminum alloy after being reheated for 1 min at a heating rate of 600 °C/min and subsequently quenched in water. The target temperature varied between 590 °C and 610 °C. The microstructure clearly exhibits the primary solid phase (white grey) and the eutectic phase (dark grey), representing the solid grains and the liquid phase that are expected to be present in the semi-solid state. The microstructure contains regularly recrystallized grains, with intragranular liquid droplets uniformly distributed and smaller in size compared to those reheated using an electrical resistance furnace. The average grain size is approximately 30 μm, and the shape factor ranges from approximately 0.82 to 0.85. Compared to those obtained by resistance furnace reheating, it can be concluded that the reheating speed affects the shape factor and the grain size, significantly. When using the resistance furnace, the shape factor reaches a steady state value of 0.85 after 60 min. However, with a higher reheating speed, the shape factor increases to 0.85 after only 1 min. Additionally, a higher reheating rate results in smaller grains that are more spherical in shape, although they still retain some facets without sharp corners. This is due to the low liquid fraction and short holding time during reheating at a higher speed, which promotes coalescence as the dominant grain coarsening mechanism, similar to resistance reheating.

### 3.2. Semi-Solid Deformation Behavior at Low Liquid Fraction

**Figure 11:**
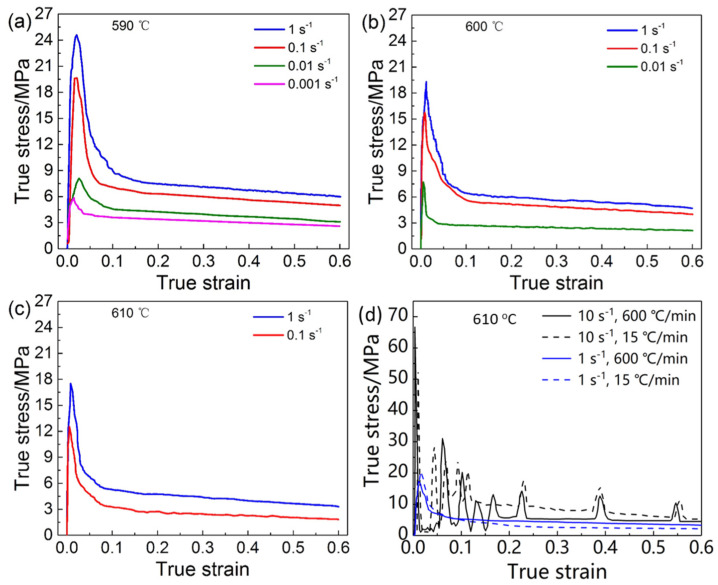
True stress−true strain curves for semi-solid 7075 alloy compressed at various strain rates. (**a**) 590 °C, (**b**) 600 °C, (**c**) 610 °C, and (**d**) 610 °C with different heating rates.

Figure 11 compares the true stress–strain curves obtained from isothermal compression tests conducted at various strain rates and temperatures, with different heating rates applied. The true stress–strain curves exhibit three distinct stages. In the first stage, the true stress rapidly reaches a peak value within a small true strain range (0–0.03). Subsequently, the stress decreases to a relatively steady value as the strain increases in the second stage. Finally, the stress value remains steady until the end of compression (the third stage). During the first stage, the slugs undergo slight deformation, causing the solid skeleton to deform and resulting in a rapid increase in stress with minimal strain. As compression continues, the solid skeletons break down and gradually form a suspension with solid agglomerates, where the solid grains are surrounded by a liquid phase. This leads to a rapid decrease in stress during the second stage. In the last stage of compression, the metallic bond is completely broken, and the material flow is driven by the motion of solid grains in the liquid phase, resulting in lower flow resistance in the steady stage.

As shown in Figure 11a–c, both the peak and steady stresses increase with the strain rate at a given temperature when the strain rate is relatively low (10^−3^ s^−1^, 10^−2^ s^−1^, 10^−1^ s^−1^, and 1 s^−1^). Additionally, an increase in temperature leads to a higher liquid fraction, resulting in a reduction in the content of interparticle contacts and a decrease in peak stress. This is because, under these strain rates, there is sufficient time for the liquid phase to flow between the solid grains during compression. However, at a high strain rate of 10 s^−1^, a unique phenomenon of repeated peak stresses occurs during compression (Figure 11d). Figure 11d compares the stress–strain curves of slugs reheated at different reheating rates and isothermally compressed at 610 °C for various strain rates. It is observed that the peak stress is larger for higher heating rates compared to lower heating rates. However, for the lower strain rate of 1 s^−1^, the peak stress decreases with a microstructure containing larger grains. This can be attributed to the different mechanisms of liquid path formation at various strain rates.

## 4. Discussion

### 4.1. The Effect of Heating Rate on the Partially Remelted Microstructure

Recrystallization is commonly defined as the formation and migration of high angle boundaries in the solid state, which occurs due to the reduction of stored energy [33]. In contrast to the conventional strain-induced recrystallization in the solid state, the recrystallization discussed in this study also involves the nucleation and growth of new equiaxed grains within elongated grains when the liquid phase is present at the high angle boundaries of the material above the solidus temperature.

In the solid state, the recrystallization temperature for 7075 Al alloy was supposed to be between 430 °C and 480 °C [34]. As shown in Figure 4, recrystallization of the extruded alloy occurred during continuous reheating from the solid state to the semi-solid state. The alloy is difficult to recrystallize until 500 °C, which may be attributed to the presence of dispersoid particles that pin the grain boundaries. Furthermore, observations have indicated that the promotion of re-crystallizing grain nucleation is facilitated by the presence of large particles that are distributed with significant intervals. Consequently, an increased density of active nucleation sites can be achieved. On the contrary, the presence of small particles that are closely spaced has been found to lower the density of active nucleation sites, thereby impeding the growth of recrystallizing grains [35]. As the material is reheated to the semi-solid state (≥550 °C), large recrystallized grains appear, as shown in Figure 4. In order to uphold stability, the elongated grains undergo a transformation into equiaxed grains with the aim of minimizing the free energy. The acceleration of the recrystallization process occurs due to the elevated diffusion rate and the abundant atomic activity experienced at high temperatures.

By comparing the grain size heated by different heating rates (Figure 4 and Figure 10), these results suggest that low heating rates lead to coarse grains. This indicates that the recrystallized grain sizes in the alloy are highly influenced by the heating rate. The larger recrystallized grain size observed with a low heating rate can be attributed to the activation of highly favored nucleation sites at an earlier stage, allowing them to have time to grow and consume neighboring potential nucleation sites, resulting in the formation of large grains. Wang et al. [36] stated that a lower reheating rate can consume a large proportion of the stored energy of dislocations by strong recovery. Smaller grains could be obtained with higher heating rates in both the solid state [25] and semi-solid state [37]. However, further investigation is required to determine the underlying reasons.

The presence of fine and globular grains in the microstructure of semi-solid billets enhances their formability. Furthermore, as shown in Figure 10, the microstructure of the semi-solid billets remains similar at different temperatures. This similarity contributes to the minimal variation in thixotropic behavior during the thixoforming process [38]. Therefore, the refined microstructure can be readily achieved across a wide range of solid fractions. The rapid heating process to achieve a fine globular semi-solid microstructure is of significant interest for industrial applications. This is because the short heating time can effectively reduce the manufacturing cost, while also improving the mechanical properties of 7075 components through grain refinement with appropriate heat treatment.

### 4.2. Thixotropic Strength Variation

The stress–strain curves depicted in Figure 11 exhibit similarities with those obtained by other researchers [39]. During the compression test, the peak stress was applied to induce the breakdown of the solid skeleton and the decohesion of grain boundaries that were not sufficiently wetted. This led to the formation of liquid paths and the initiation of thixotropic flow. The peak stress can be referred to as thixotropic strength. The Voigt model was used to understand the behavior of semi-solid alloys. This model includes a saturated solid skeleton and a concentrated suspension branch. By employing this model, researchers aimed to elucidate the deformation behavior of these alloys [40]. Favier and Atkinson [41] proposed a response model for semi-solid alloys that combines elasticity and viscoplasticity. They attributed the elasticity to the saturated solid skeleton, while the viscoplasticity was linked to the deagglomeration process of the saturated solid skeleton. The peak stress experienced by semi-solid alloys during compression tests, as predicted by the Voigt model, is influenced by both the elastic and viscoplastic responses. The variations in peak stress can be attributed to the microstructural characteristics of semi-solid slugs, such as solid fraction, shape factor of grains, and the liquid phase distribution. It is important to note that the strength of the skeleton predominantly depends on the solid fraction. As the semi-solid temperature increases, the solid fraction decreases, resulting in a decrease in skeleton strength. Consequently, samples tested at higher semi-solid temperatures demonstrate lower peak stress (Figure 11a–c). These findings highlight the importance of understanding microstructural properties when studying the mechanical behavior of semi-solid alloys. By considering factors such as solid fraction and temperature, researchers can gain insight into the viscoplastic behavior of these materials. This knowledge can contribute to advancements in designing and engineering semi-solid alloys with the desired mechanical properties.

**Figure 12:**
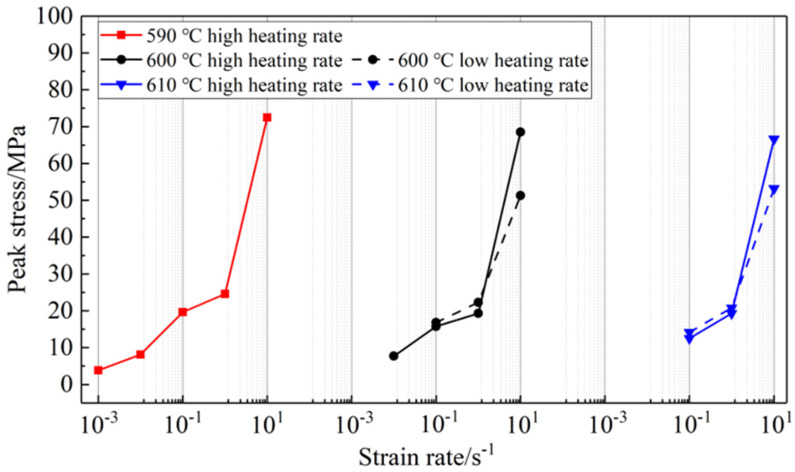
Variations of peak stress vs. strain rates at various temperatures.

Figure 11d also demonstrates the impact of grain size on peak stress. Additionally, Figure 12 displays the variations of peak stress VS. strain rates at various temperatures. At lower strain rates, slugs with larger grains, which are reheated at a low heating rate, exhibit higher peak stress compared to those with fine grains. This is because larger grains, with a similar low liquid fraction, result in a higher degree of connectivity and stronger skeleton strength. Consequently, the peak stress increases with increasing grain size for a similar liquid fraction. However, at high strain rates, slugs with larger grains have a lower peak stress than those with fine grains. This could be attributed to the strong shear forces causing the liquid to squeeze out and gather among the coarse solid particles, resulting in a lower peak stress compared to fine solid particles. Semi-solid compression may also lead to agglomeration and deagglomeration, particularly under high strain rates, which can cause fluctuations in the stress–strain curves. Shan and Luo [42] also observed similar results in their investigation of the compression behavior of semi-solid Mg alloy. They reported that the multiple peaks of the stress–strain curves are attributed to the aggregation of solid grains and the sudden disintegration of solid aggregates by strong shear action.

## 5. Conclusions

This study investigated the impact of temperature and heating rate on the microstructure evolution of hot-deformed 7075 Al alloy during reheating and holding in the semi-solid state. Additionally, isothermal semi-solid compression tests were conducted on slugs with different microstructures. The main conclusions are as follows:

1. Microstructures with globular solid grains surrounded by liquid phase can be obtained by semi-solid isothermal holding. The coarsening rate constant increases with higher temperatures and longer holding times.
2. The grain sizes in the 7075 aluminum alloy, obtained through the semi-solid reheating and holding process, are highly influenced by the heating rate in the final recrystallization stage. Higher heating rates were found to be beneficial in achieving finer grains, which in turn can improve the formability of semi-solid billets.
3. When the semi-solid 7075 alloy is heated within the temperature range of 590 to 610 °C, it exhibits thixotropic behavior during compression. The microstructure and its micromechanical response during deformation play crucial roles in the thixotropic flow. The agglomeration and deagglomeration behaviors of solid grains result in variations of peaks, which depend on grain size, solid fraction, and strain rate.
4. In this study, the thixoforged samples are relatively simple and have a single deformation direction. However, for further industrial application, it is important to investigate the optimization of the heating rate on the microstructure evolution and mechanical properties of bulk complex parts.

## Figures and Tables

**Table 1:** Chemical composition of 7075 aluminum alloy.

Element	Zn	Mg	Cu	Fe	Cr	Mn	Si	Al
wt%	5.8	2.45	1.58	0.42	0.225	0.17	0.36	Balanced

**Table 2:** EDS results of hot-extruded 7075 alloy (at.%).

**Area**	**Al**	**Zn**	**Mg**	**Cu**	**Si**	**Fe**	**Mn**	**Cr**	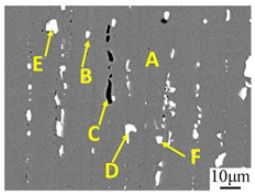
A	93.9	2.9	2.6	0.6					
B	56.8	2.9	20.1	20.2	0.1				
C	27.2	0.5	43.9		28.4				
D	80.1	1.9		4.1		12.6	1.0	0.3	
E	75.9	0.6		2.3	4.9	10.9	2.4	2.9	
F	84.7	2.2	1.2	2.8		8.1	08	0.2	

**Table 3:** EDS analysis of semi-solid 7075 aluminum alloy corresponding to Figure 5.

Element		Al	Zn	Mg	Cu	Fe	Cr	Mn
Point A	wt%	89.84	6.53	2.3	1.33			
	at%	93.92	2.82	2.67	0.59			
Point B	wt%	46.37	6.9	1.39	45.34			
	at%	66.23	4.07	2.2	27.5			
Point C	wt%	62.47	8.38	6.60	14.34		8.21	
	at%	74.73	4.14	8.76	7.27		5.10	
Point D	wt%	64.84	3.79		11.15	17.81		2.41
	at%	80.12	1.93		5.85	10.63		1.46

## Data Availability

The data that support the findings of this study are available from the corresponding author upon reasonable request.
